# EurSafety Health-net: development of an EURegional infection control quality certificate for nursing homes

**DOI:** 10.1186/1753-6561-5-S6-P164

**Published:** 2011-06-29

**Authors:** A Eikelenboom-Boskamp, A Haenen, R Koopmans, A Voss

**Affiliations:** 1 Medical Microbiology , Radboud University Medical Centre, Netherlands; 2 Medical Microbiology and Infectious Diseases, Canisius Wilhelmina Hospital, Nijmegen, Netherlands; 3 Epidemiology and Surveillance, National Institute for Public Health and the Environment, Bilthoven, Netherlands; 4 Primary and Community Care, Radboud University Medical Centre, Nijmegen, Netherlands

## Introduction / objectives

As part of the EurSafety Health-net project, a cross-border German-Dutch healthcare project to reduce HAIs, and in cooperation with regional specialists in care for the elderly, we developed an infection control quality improvement program for nursing homes. Aim of this multi-year project is to implement a complete infection control program for nursing homes in a step-by-step fashion.

## Methods

A multi-step plan, each consisting of 10 measures, was developed to improve infection control practices in nursing homes. The audit was done by an EurSafety auditteam. Organizations reaching 5 of the 10 criteria receive a certificate and have up to 3 years to fullfil the remaining criteria, before advancing to the next step. Participation and certification are depicted on a website accessible for the public.

## Results

The first certificates have been distributed in January 2011 to 27 institutions in the Nijmegen region. The initial ten criteria consisted of structure and process criteria, as well as HAI-prevalence and UTI-incidence studies. The poster will describe the criteria in detail.

## Conclusion

Due to the frequent exchange of patients between healthcare facilities, Infection control programs in long-term care facilities become increasingly important. The EurSafety quality program stimulates LTCF’s to structurally improve their performance, while the certificate gives visibility of their efforts to all stakeholders.

## Disclosure of interest

None declared.

